# Reliability of *KRAS* mutation testing in metastatic colorectal cancer patients across five laboratories

**DOI:** 10.1186/1756-0500-5-196

**Published:** 2012-04-25

**Authors:** Heather Spencer Feigelson, Katrina AB Goddard, Monique A Johnson, Kellyan C Funk, Alanna Kulchak Rahm, Tia L Kauffman, Dhananjay A Chitale, Loic Le Marchand, C Sue Richards

**Affiliations:** 1 Institute for Health Research Legacy Highlands, Kaiser Permanente Colorado, Suite 300, P.O. Box 378066, Denver, CO, 80237-8066, USA; 2 Kaiser Permanente Northwest, Center for Health Research, 3800 N. Interstate Avenue, Portland, OR, 97227, USA; 3 Molecular and Medical Genetics, Oregon Health & Science University, 3181 S.W. Sam Jackson Park Road, Portland, OR, 97239-3098, USA; 4 Department of Pathology and Laboratory Medicine, Henry Ford Hospital, 2799 W. Grand Blvd., Detroit, MI, 48202, USA; 5 University of Hawaii Cancer Center, 677 Ala Moana Boulevard, Suite 901, Honolulu, HI, 96813, USA

**Keywords:** KRAS, Colorectal cancer, EGFR, Laboratory error

## Abstract

**Background:**

Mutations in the *KRAS* gene are associated with poor response to epidermal growth factor receptor inhibitors used in the treatment of metastatic colorectal cancer. Factors influencing *KRAS* test results in tumor specimens include: tumor heterogeneity, sample handling, slide preparation, techniques for tumor enrichment, DNA preparation, assay design and sensitivity. We evaluated comparability and consistency of *KRAS* test results among five laboratories currently being used to determine *KRAS* mutation status of metastatic colorectal cancer specimens in a large, multi-center observational study.

**Findings:**

Twenty formalin-fixed paraffin-embedded human colorectal cancer samples from colon resections previously tested for *KRAS* mutations were selected based on mutation status (6 wild type, 8 codon 12 mutations, and 6 codon 13 mutations). We found good agreement across laboratories despite differences in mutation detection methods. Eighteen of twenty samples (90%) were concordant across all five labs. Discordant results are likely not due to laboratory error, but instead to tumor heterogeneity, contamination of the tumor sample with normal tissue, or analytic factors affecting assay sensitivity.

**Conclusions:**

Our results indicate commercial and academic laboratories provide reliable results for the common *KRAS* gene mutations at codons 12 and 13 when an adequate percentage of tumor cells is present in the sample.

## Findings

### Background and hypothesis

Anti-epidermal growth factor receptor (EGFR) monoclonal antibodies are approved for the treatment of metastatic colorectal cancer (CRC). However, these anti-EGFR therapies do not benefit patients whose tumors harbor a *KRAS* mutation [1]. Genetic testing for the presence of *KRAS* mutations has been recommended to guide treatment for these patients [2].

Several factors can influence *KRAS* mutation testing results in CRC specimens [3-5]. The purpose of this study was to evaluate comparability and consistency of clinical *KRAS* test results among laboratories used to determine *KRAS* mutation status in a large multi-center study. Three commercial laboratories (Genzyme, Clarient, Quest Diagnostics), one clinical academic laboratory (Henry Ford Health System), and one academic research laboratory (Molecular and Medical Genetics, Oregon Health and Science University) were contracted to analyze *KRAS* mutation status for comparison with previous clinical results. While all five laboratories are Clinical Laboratories Improvement Act (CLIA) certified, they have different sample preparation and mutation detection methods. Our aim was not to certify these laboratories, but to ensure that we could combine data from previously tested clinical samples in our research study.

### Methods

Twenty surgical specimens from colon resections were used; eighteen specimens were adenocarcinomas, two were carcinomas. Blocks were reviewed by a pathologist to determine whether the samples were of sufficient quality and quantity for testing, then the samples were de-identified and slides were prepared per individual laboratory specifications. Our intention was to replicate routine sample testing of clinical specimens as much as possible, so sample handling and shipping procedures varied slightly by laboratory. All laboratories used microdissection for tumor enrichment when necessary, but mutation detection methods differed. Methods for each laboratory are described in Table 1.

**Table 1:** Specimen requirements and assay specifications of KRAS genotyping by laboratories

	Lab #1 (Sequencing)	Lab #2 (Sequencing)	Lab #3 (Sequencing)	Lab #4 (Primer Extension)	Lab #5 (Real Time PCR)
Specimen Requirements	Preferred sample type*: Slides from FFPE block 1 H&E stained slide sections with tumor circled; 4 matching unstained slides, 10 microns each.	Preferred sample type: Archival FFPE or frozen surgical biopsies confirmed to contain >50% tumor by a surgical pathologist. 1 H&E slide; 5 unstained sections, 10 microns each.	Preferred sample type: FFPE tissue 6 unstained sections, 10 microns each.	Preferred sample type: Pre-cut slides from FFPE. Send all slides within 5–7 days of cutting. Air dry. Do not oven dry. Store specimen at room temperature (20–23.5°C). 5 unstained sections, 7 microns each	Preferred sample type: FFPE block, unstained slides, or fresh snap frozen biopsy 5 unstained sections, 7 microns each
Genotyping	Method: PCR amplification followed by Direct Sanger sequencing (Big Dye v. 1.1) Detected mutations: KRAS codons 12 and 13	Method: PCR amplification followed by standard bidirectional sequencing on ABI 3100. Detected mutations: KRAS codons 12 and 13	Method: PCR amplification followed by sequencing. Detected mutations: KRAS codons 12, 13 and 61	Method: Single nucleotide primer extension with fragment analysis by capillary electrophoresis using a modified SNaPshot assay. Detected mutations: KRAS codons 12 and 13	Methods are propietary: qualitative real time PCR Detected mutations: KRAS codons 12 and 13
Lower Limit of Detection	20% when ≥ 40% tumor cells present	20%	15-20%	10% when ≥ 2% tumor cells present	1-5%

*For this study, slides prepared from Formalin-fixed paraffin embedded (FFPE) blocks were sent to each lab.

*KRAS* test results were compared across labs, and discrepancies were evaluated further. This study was approved by the Institutional Review Boards (IRB) at Kaiser Permanente Colorado and Kaiser Permanente Northwest (the Oregon Health and Science University IRB ceded authority to Kaiser Permanente Northwest).

### Results

Twenty formalin-fixed paraffin-embedded (FFPE) CRC samples previously tested clinically for *KRAS* mutations by sequencing were selected based on mutation status (6 wild-type samples, 8 with codon 12 mutations, and 6 with codon 13 mutations) from two study sites (Kaiser Permanente Colorado and Northwest). Patients ranged in age from 46–85 years; specimens were collected between 2005–2009. We found good agreement in *KRAS* test results with prior clinical results despite differences in mutation detection methods (Table 2). Eighteen of twenty samples (90%) were concordant across all five laboratories, and the mutation type was always consistent.

**Table 2:** Results of *KRAS* testing by five CLIA-certified laboratories

Sample ID	Clinical Result*	Sequencing Lab 1	Sequencing Lab 2	Sequencing Lab 3	Primer Extension Lab 4	Real-time PCR Lab 5
1	WT**	WT	WT	WT	WT	WT
2	WT	WT	WT	WT	WT	WT
3	WT	WT	WT	WT	WT	WT
4	WT	WT	WT	WT	WT	WT
5	WT	WT	WT	WT	WT	WT
6	WT	WT	G12D	G12D	WT	WT
7	G12V	G12V	G12V	G12V	G12V	G12V
8	G12D	G12D	G12D	G12D	G12D	G12D
9	G12V	G12V	G12V	G12V	G12V	G12V
10	G12S	G12S	G12S	G12S	G12S	G12S
11	G12V	G12V	G12V	G12V	G12V	G12V
12	G12C	G12C	G12C	G12C	G12C	G12C
13	G12V	G12V	G12V	G12V	G12V	G12V
14	G12D	WT^†^	G12D	G12D	G12D	G12D
15	G13D	G13D	G13D	G13D	G13D	G13D
16	G13D	G13D	G13D	G13D	G13D	G13D
17	G13D	G13D	G13D	G13D	G13D	G13D
18	G13D	G13D	G13D	G13D	G13D	G13D
19	G13D	G13D	G13D	G13D	G13D	G13D
20	G13D	G13D	G13D	G13D	G13D	G13D

*Samples were selected for testing based on these prior clinical results.

^**^WT: wildtype; G12D: p.Gly12Asp; G12V: p.Gly12Val; G12S: p.Gly12Ser;

G13D: p.Gly13Asp.

^†^This laboratory did see some evidence that the sample had a mutation, but was below the confidence threshold. This specimen showed tumor enrichment of approximately 40%, which is at the lower level of detection for this laboratory.

One laboratory reported a wild-type result for sample 14 which was actually a p.Gly12Asp (G12D) mutation. While this sample was confirmed to contain an acceptable 40% tumor cells, tumor heterogeneity in this sample may have resulted in the variant being present below the pre-determined 20% threshold. A very small electropherogram peak indicating a c.35 G > A change was visible by sequencing indicating p.Gly12Asp mutation, but was not reported because it was below acceptable level of confidence per laboratory protocol.

We also found a discrepancy in sample 6. The initial clinical result was wild-type; two labs reported a mutation in exon 12 p.Gly12Asp, and three labs were consistent with the clinical results (wild-type). We evaluated this discrepancy first by sending additional slides (from the same tumor block) to the two laboratories that reported the mutation. These slides were assigned a new study number to blind the laboratory to the re-testing. Laboratory #2 found the same result (p.Gly12Asp) in the second set of slides, but there was not enough tumor tissue for laboratory #3 to reliably genotype. Next, we used a second FFPE block from the same patient to send a third set of slides with a new study number to laboratories 1–3. Results from this block were concordant at all three laboratories (*KRAS* wild-type). Finally, we asked laboratories #1 and #2 to “swap” aliquots of the extracted DNA from their original sample 6 FFPE slides. This re-analysis confirmed the initial (discrepant) results at each laboratory. Thus, we conclude that the laboratory results, while different, are accurate for the sample of tissue received at each laboratory. The discrepant results could be due to either true tumor heterogeneity or contamination of the tumor sample with normal tissue. We cannot conclusively determine which of these two scenarios is responsible for our observed results, nor eliminate the possibility that a laboratory error resulting in sample mix-up lead to the discrepant results.

### Discussion

We found high concordance of *KRAS* test results with previously received clinical results across five laboratories, despite differences in laboratory methods. The discordant results observed in two samples are most likely due to sample characteristics rather than to laboratory error. Our study focused only on mutations in codons 12 and 13 of *KRAS*. These are the most common mutations and are often the only mutations targeted in clinical testing. However, other mutations may have clinical implications and were not included in our study [6,7].

We limited our study to samples from colon resections. Samples with smaller volume, such as from metastatic sites or biopsy, may not perform as reliably as colectomy specimens if they contain only a small percentage of tumor cells. Several new methods of mutation detection have been reported [3,8-13] and may be better suited to samples with a low percentage of tumor cells.

Our results are in agreement with previous studies [8-12], including a recent report by Oliner et al. [13] who evaluated five commercial laboratories, one of which (Genzyme) was also included in our study. They tested forty FFPE samples from several tissue procurement providers, whereas our samples were obtained from colectomies performed at our own clinic facilities, previously tested, and used to guide clinical care. Because we were able to select our samples based on mutation status, and thus oversample for the *KRAS* mutations of interest, our estimate of the agreement across laboratories corresponds to an estimate from an effectively larger sample size. It is reassuring that, while both studies evaluated different commercial laboratories and used slightly different methodologies, both found good agreement across testing facilities.

The commercial and academic-based laboratories included in this study provide reliable test results for common mutations in the *KRAS* gene from samples with an adequate percentage of tumor cells. Discrepancies observed are likely due to either tumor heterogeneity, or contamination of the tumor sample with normal tissue. Both of these sources of variability are likely to be encountered in the clinical setting, and may have important consequences for treatment decisions.

## Abbreviations

CLIA, Clinical Laboratories Improvement Act; CRC, colorectal cancer; EGFR, epidermal growth factor receptor; FFPE, formalin-fixed paraffin-embedded; H&E, hematoxylin and eosin; IRB, Institutional Review Board; WT, wild-type.

## Competing interests

The authors declare that they have no competing interests.

## Authors’ contributions

HSF conceived of the study, participated in its design and drafted the manuscript. KABG designed the larger study under which this research was carried out and contributed to the design of this study. MAJ participated in the laboratory analysis. KCF coordinated collection of samples for analysis and collated results. AKR managed the study, participated in the study design, and participated in manuscript preparation. TLK managed the larger study and assisted in coordinating sample collection and results. DAC participated in the laboratory analysis of samples. LLM conceived of the study and participated in its design and manuscript preparation. CSR participated in laboratory analysis, interpretation of results, and participated in manuscript preparation. All authors read and approved the final manuscript.
